# New Ground in Antifungal Discovery and Therapy for Invasive Fungal Infections: Innovations, Challenges, and Future Directions

**DOI:** 10.3390/jof10120871

**Published:** 2024-12-15

**Authors:** Gustavo A. Niño-Vega, Leonardo Padró-Villegas, Everardo López-Romero

**Affiliations:** Departamento de Biología, División de Ciencias Naturales y Exactas, Campus Guanajuato, Universidad de Guanajuato, Noria Alta s/n, col. Noria Alta, Guanajuato C.P. 36050, Mexico; l.padrovillegas@ugto.mx

**Keywords:** antifungal therapy, drug resistance, fungal infections, novel antifungals, nanoparticles, natural products

## Abstract

This review explores current advancements and challenges in antifungal therapies amid rising fungal infections, particularly in immunocompromised patients. We detail the limitations of existing antifungal classes—azoles, echinocandins, polyenes, and flucytosine—in managing systemic infections and the urgent need for alternative solutions. With the increasing incidence of resistance pathogens, such as *Candida auris* and *Aspergillus fumigatus*, we assess emerging antifungal agents, including Ibrexafungerp, T-2307, and N′-Phenylhydrazides, which target diverse fungal cell mechanisms. Innovations, such as nanoparticles, drug repurposing, and natural products, are also evaluated for their potential to improve efficacy and reduce resistance. We emphasize the importance of novel approaches to address the growing threat posed by fungal infections, particularly for patients with limited treatment options. Finally, we briefly examine the potential use of artificial intelligence (AI) in the development of new antifungal treatments, diagnoses, and resistance prediction, which provides powerful tools in the fight against fungal pathogens. Overall, we highlight the pressing need for continued research to advance antifungal treatments and improve outcomes for high-risk populations.

## 1. Introduction

Fungi are key players in ecosystems, serving as decomposers, symbionts, and pathogens. Beyond their ecological role, fungi are critical to the industrial sector, including biotechnology, pharmaceuticals, and food production. They have been used to develop antibiotics and play a role in fermentation processes. On the other hand, fungal pathogens pose significant health risks. Despite their relatively small numbers compared to bacterial pathogens, fungi cause significant morbidity and mortality, especially in hospital settings [1].

Fungi comprise a distinct kingdom of eukaryotic organisms, diverging from animals roughly 1.3 billion years ago, with their own evolutionary path that sets them apart from plants and animals [2]. Recent estimates suggest there may be between 2.2 and 12 million fungal species, but fewer than 5% of these species have been formally described [3,4]. This wide range of estimated fungal species takes into consideration the unculturable fungi detected through environmental DNA (eDNA) sequencing but not grown in laboratory conditions, referred to as “dark matter taxa” [2,3]. These taxa constitute a significant portion of fungal biodiversity, contributing to the underestimation of global fungal diversity. High-throughput sequencing techniques have brought to light many dark matter taxa, which are essential to understanding the true extent of fungal diversity [2]. Their inclusion in phylogenetic analyses helps to refine evolutionary models and improves species classification [2].

Fungal infections have become an increasing health concern, especially in immunocompromised patients, such as those with HIV, cancer, or organ transplants, who are particularly vulnerable to these infections and present high morbidity and mortality rates, often exceeding 40%, even with treatment [1,4,5]. Only about 300 fungal species have been identified as pathogenic to humans [4]. These infections range from superficial skin infections to life-threatening invasive diseases. Existing treatments are limited to a handful of antifungal classes (Table 1), with most therapies targeting only a few fungal species, such as *Candida*, *Aspergillus*, and *Cryptococcus*. While these drugs are effective, they are limited by toxicity, drug–drug interactions, limited clinical efficacy, and the emergence of antifungal resistance, particularly in species such as *Candida auris* and *Aspergillus fumigatus*, complicating treatment strategies and highlighting the need for new antifungal agents [4,6,7]. In the present review, we discuss the recent advances in antifungal strategies focusing on improving efficacy and reducing resistance, including the production of novel compounds, combining antifungals with different mechanisms of action to improve efficacy and reduce the risk of resistance (combination therapy), drug repurposing, and the use of nanoparticles as conveyors of antifungals, thus enhancing their efficacy.

## 2. New Developments in Antifungal Agents

### 2.1. Current Antifungal Therapies

There are four major classes of antifungal agents available for the treatment of systemic fungal infections (Table 1).

Azoles: Azoles inhibit ergosterol synthesis by targeting essential fungal lanosterol 14α-demethylase (CYP51 enzymes), disrupting cell membrane integrity. Examples include fluconazole, itraconazole, and voriconazole [5,8,9], as well as newer azoles, such as opelconazole and oteseconazole [7]. Azoles are widely used due to their broad-spectrum activity, particularly for treating *Candida*, *Cryptococcus*, and *Aspergillus* infections. However, long-term use can lead to resistance, particularly in *Candida* species. Azoles also have significant drug interactions due to their effects on cytochrome P450 enzymes [4].

Echinocandins (e.g., Caspofungin, Micafungin, and Anidulafungin): Echinocandins are lipopeptides that inhibit fungal growth as they block β-(1,3)-glucan synthesis, an essential component of the fungal cell wall [10], and are effective against *Candida* and *Aspergillus* species, particularly azole-resistant strains [5,8]. Echinocandins are ineffective against some other fungal species, such as *Cryptococcus neoformans*, and resistance is emerging in some strains [6].

Polyenes (e.g., Amphotericin B): Polyenes bind to ergosterol in fungal cell membranes, creating pores that lead to cell death. They are effective against various fungi, including *Aspergillus*, *Cryptococcus*, and *Candida*. However, they are highly toxic, particularly to the kidneys, leading to severe side effects, including nephrotoxicity. Liposomal formulations reduce toxicity but are expensive [4]. Lipid-based encochleated amphotericin B is more stable than liposomes, more resistant to oxidation and enzyme degradation, and enables the slow release of the drug [7].

Flucytosine: This pyrimidine analog interferes with fungal RNA and DNA synthesis after the cell’s conversion to 5-fluorouracil (5-FC). It is commonly used in combination with amphotericin B, followed by fluconazole consolidation, to treat *Cryptococcus* infections. It is also active against candidiasis and chromoblastomycosis. It presents rapid development of resistance when used as monotherapy and has a narrow antifungal spectrum, as well as bone marrow toxicity [4,8]. It has recently been reported that mutations in the FUR1 and FCY1 genes involved in the pyrimidine salvage pathway in *C. neoformans* resulted in the absence or reduction of resistance to 5-FC. Genetic transformations demonstrated the relationship between these mutations and drug resistance [11].

### 2.2. New Antifungal Drugs

**Figure 1:**
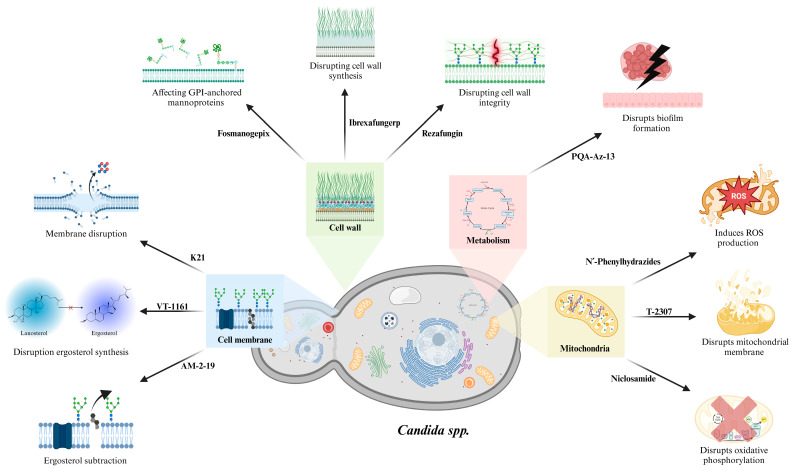
Mechanisms of action of novel antifungal drugs.

The development of new antifungals for human mycoses is a complex process due to several factors: (1) Limited Molecular Targets: Fungi are eukaryotes, like their human hosts; thus, there are few unique targets for selective antifungal drug development [5,8]. (2) Drug Permeability: Fungal cell walls and membranes can hinder drug penetration, reducing the efficacy of many potential antifungal agents [5]. (3) Resistance Development: Even with novel drugs, resistance can develop rapidly, as seen with azoles and echinocandins [8]. Considering the challenges associated with the limitations of current therapies, there is an urgent need for new antifungals with novel mechanisms of action and improved safety profiles. Several promising candidates are in various stages of development, including repurposed drugs and new antifungal classes (Figure 1, Table 2):

Compound K21: K21 is a silica quaternary ammonium compound (SiQAC) with tetraethoxysilane (TEOS), which has been used as a wide-spectrum anti-antiviral and anti-microbial compound [6,12,13,14]. It primarily acts through membrane disruption, leading to rapid fungal cell death. The compound demonstrated significant antifungal activity against fluconazole-susceptible and -resistant *Candida* species, including *Candida albicans*, *C. glabrata*, and *C. dubliniensis* [6]. Scanning electron microscopy (SEM) and transmission electron microscopy (TEM) studies revealed that K21 induces morphological changes, such as the formation of extracellular vesicles, membrane blebbing, and eventual cell lysis, indicating a potent membrane damage mechanism. K21’s positive charge attracts negatively charged fungal cells, allowing the compound’s long carbon chains to penetrate and rupture the cell walls. K21 demonstrated fungicidal activity within two hours, killing 99.9% of fluconazole-resistant *Candida* isolates. It exhibited synergistic effects when combined with fluconazole, particularly in strains like *C. dubliniensis* and *C. tropicalis* [6].

Olorofim (F9013_118): A first-in-class antifungal agent from the orotomide class, originally discovered through in vitro screening for antifungal properties of a library of small molecules against *Aspergillus fumigatus* [15]. It selectively inhibits dihydroorotate dehydrogenase (DHODH)—an enzyme crucial for the de novo pyrimidine biosynthesis pathway in fungi—leading to a reduction in pyrimidine production, affecting various cellular processes [15,16]. Pyrimidines, such as uracil, cytosine, and thymine, are required for nucleic acid synthesis, while the pyrimidine derivative UTP is needed to form essential cell wall components, such as β-1,3-glucan and chitin. In *A. fumigatus*, olorofim was shown to induce significant morphological changes, including increased chitin content in the cell wall (likely as a compensatory response to the inhibited synthesis of β-1,3-glucan), increased septation, vacuolar enlargement, and inhibition of mitosis [16,17]. The increased septation and reduced interseptal distance suggest an effort by the fungus to survive the disrupted cell wall integrity [17]. Additionally, vacuolar swelling—a phenomenon associated with autophagic processes—potentially indicates stress-induced cell death pathways. Its ability to interfere with fundamental processes, such as pyrimidine biosynthesis, and its unique action on cell wall remodeling and septation positions it as a strong candidate for treating drug-resistant fungal pathogens. Olorofim is currently in phase II clinical trials, with encouraging results in terms of its efficacy and safety against invasive fungal infections caused by *Aspergillus fumigatus* and other difficult-to-treat filamentous fungi; however, it has no effect against *Candida albicans* and other *Candida* species [16].

VT-1161 (otesoconazole): A tetrazole antifungal designed to target lanosterol 14α-demethylase (LDM), a key component of fungal cell membranes [18,19]. By inhibiting LDM, VT-1161 disrupts ergosterol production, leading to compromised membrane integrity and fungal cell death. Similar to triazole drugs, VT-1161 functions by binding to the heme iron within the LDM active site, disrupting the demethylation of lanosterol—a crucial step in ergosterol biosynthesis. The compound’s tetrazole ring provides a slightly lower affinity towards the fungal enzyme than the triazole drugs but also allows for greater selectivity than human LDM homologs. Structural studies have shown that VT-1161 forms a hydrogen bond network involving a water molecule and key active site residues in *S. cerevisiae* LDM, such as Y140, strengthening its binding to the fungal enzyme. VT-1161 has potent activity against *Candida glabrata* and *Candida krusei*, which are often resistant to traditional azole antifungals, such as fluconazole. The drug’s effectiveness against *Candida albicans* strains is dramatically reduced with overexpression of drug efflux pumps, such as CaCdr1 and CaMdr1. Azole resistance mechanisms, including LDM overexpression and mutations in the LDM active site, can also reduce the efficacy of VT-1161. Specific mutations, such as Y140F and Y140H, in the LDM active site alter its interaction with the tetrazole ring, resulting in decreased binding affinity and higher resistance [19]. In a recent study, it was demonstrated that VT-1161 was a potent inhibitor of mono-microbial biofilms formed by *C. albicans*, *Klebsiella pneumoniae*, and *Staphylococcus aureus*, as well as dual biofilms of *C. albicans*/*K. pneumoniae* and *C. albicans*/*Staphylococcus aureus* [20]. Furthermore, it showed low toxicity in human cell lines and *Caenorhabditis elegans*. On this basis, it was proposed as a good option to prevent or treat mono- or poly-microbial biofilms [20].

T-2307: T-2307 is a novel arylamidine compound with potent antifungal activity against *Candida* spp., *Cryptococcus* spp., and *Aspergilluis* spp. [21,22,23,24,25]. T-2307 accumulates in *C. albicans* yeast cells via high-affinity spermine and spermidine transporters [23] and selectively targets fungal cells by disrupting the mitochondrial membrane potential [26], thus inhibiting the respiratory chain complexes III and IV in *C. albicans* yeast without significantly affecting mammalian mitochondrial membranes [26]. The disruption of mitochondrial activity leads to a collapse of fungal cell energy production, resulting in cell death [25,26]. T-2307 has shown remarkable efficacy against *Candida tropicalis*, a pathogen known for its biofilm formation, which contributes to its virulence and drug resistance. The compound demonstrated a minimum inhibitory concentration (MIC) of 0.005 µg/mL against *C. tropicalis* clinical isolates, a value significantly lower than that of fluconazole. It inhibited biofilm formation at sub-MIC levels, with a 70% reduction in biofilm biomass at concentrations as low as 0.0025 µg/mL (0.5× MIC). Moreover, T-2307 eradicated 70% of mature biofilms at 20× MIC. Additionally, T-2307 significantly downregulated key virulence-related genes in *Candida tropicalis*, such as *HWP1* (which is involved in hyphal growth), while upregulating stress-related genes, like *ERG11* (associated with ergosterol biosynthesis). This dual impact on biofilm inhibition and gene expression suggests that T-2307 targets biofilm formation and cellular resilience mechanisms [25].

In a *Galleria mellonella* infection model, T-2307 demonstrated significant antifungal activity. Both pre- and post-infection treatments with T-2307 increased the survival rate of larvae infected with *C. tropicalis*. The compound was non-toxic to the larvae at concentrations up to 2× MIC, showing a significant reduction in survival at 4× MIC. T-2307 showed low cytotoxicity on human prostate epithelial cells (PNT1A), maintaining over 80% cell viability even at high concentrations (0.1 µg/mL). Additionally, T-2307 reduced the secretion of pro-inflammatory cytokines (IL-6 and IL-10) in *Candida*-infected epithelial cells, highlighting its potential anti-inflammatory benefits during fungal infection. Its mode of action and low cytotoxicity make it a promising candidate for treating invasive fungal infections, such as those resistant to conventional antifungals [25].

Ibrexafungerp (formerly SCY-078 and MK-3118): Marketed as Brexafemme, this triterpenoid antifungal drug is the first oral β-(1,3)-D-glucan synthase inhibitor [27,28]. The FDA has approved it for the treatment of vulvovaginal candidiasis (VVC) and recurrent vulvovaginal candidiasis (RVVC) [28,29]. Its oral route of administration makes it a more convenient alternative to echinocandins requiring intravenous delivery. Ibrexafungerp inhibits β-(1,3)-D-glucan synthases, which weakens the fungal cell wall and leads to cell lysis [27]. While its mechanism is similar to that of echinocandins (i.e., by binding and inhibiting the activity of the β-(1,3)-D-glucan synthases), ibrexafungerp appears to interact with the enzyme at a different site [30], reducing the risk of cross-resistance. Indeed, it remains effective against *Candida* strains that harbor FKS mutations, which confer resistance to echinocandins [30]. The drug exhibits broad-spectrum fungicidal activity against numerous *Candida* species, including *C. albicans*, *C. glabrata*, *C. parapsilosis*, *C. krusei*, and *C. tropicalis* [31]. Its minimum inhibitory concentration (MIC) is particularly low in acidic environments, such as those found in vaginal tissues, which enhances its efficacy in treating vulvovaginitis [32]. It has about 50% oral bioavailability, reaching maximum plasma concentrations within 4 to 6 h. It is highly protein-bound (>99%), and animal studies have indicated that ibrexafungerp achieves nine-fold higher concentrations in vaginal tissues compared to blood [28]. It is metabolized primarily via hepatic CYP3A4, and its elimination half-life is around 20 h. Most of the drug is excreted via feces, with minimal urinary excretion. It has demonstrated clinical efficacy in two phase 3 studies, VANISH 303 and VANISH 306, which evaluated its use in treating acute VVC. In both studies, ibrexafungerp showed superior clinical response compared to the placebo, with minimal adverse effects and mostly mild gastrointestinal symptoms [28,32].

Fosmanogepix (FMGX): FMGX is a prodrug that is metabolized into its active form, manogepix (MGX), which targets the fungal Gwt1 enzyme involved in glycosylphosphatidylinositol (GPI) biosynthesis, essential for fungal cell wall integrity and pathogenicity [33,34,35]. This enzyme catalyzes the inositol acylation of GlcN-PI, which is crucial for the localization and function of GPI-anchored mannoproteins in fungal cell walls [33,36]. Inhibition of Gwt1 prevents the maturation and proper localization of these mannoproteins, leading to impaired cell wall integrity, reduced hyphal formation, and decreased virulence in fungi [33]. In vitro, it has been shown to be effective against a wide range of pathogens, including fluconazole- and echinocandin-resistant strains of *Candida* spp. and azole-resistant strains of *Aspergillus fumigatus*, and is particularly effective against difficult-to-treat pathogens such as *Scedosporium*, *Fusarium*, and *Lomentospora prolific* [37,38]. Clinical trials have shown that fosmanogepix is highly bioavailable, with oral and intravenous formulations offering flexibility in treatment. FMGX has demonstrated significant in vivo efficacy in animal models of disseminated infections caused by *Candida* species, including *C. albicans*, *C. glabrata*, and *C. auris*, as well as in pulmonary infection models of *Aspergillus fumigatus* and *A. flavus* [34]. The drug also effectively treated *Scedosporium*, *Fusarium*, and *Rhizopus* infections, with survival rates and reduced fungal burden in various tissues, including the lungs, brain, and kidneys [39,40]. FMGX has a low propensity for resistance, with studies showing limited cross-resistance with other antifungal drug classes, such as azoles and echinocandins. Resistance mutations identified in Gwt1 are rare, and mutations that do arise are linked to reduced susceptibility in a small subset of clinical isolates [41]. The drug has shown a favorable safety and tolerability profile in clinical trials, with limited drug–drug interactions and minimal adverse effects [35].

Rezafungin: Formerly CD101, rezafungin is a next-generation echinocandin [42]. As a member of the echinocandin class, it is designed to overcome the limitations of earlier echinocandins, such as their limited half-life and stability [43]. It is notable for its enhanced pharmacokinetics, allowing for once-weekly dosing, and its potential for both intravenous and alternative routes of administration, including subcutaneous and topical forms [44,45]. As with any echinocandin, rezafungin works by inhibiting the enzyme β-(1,3)-D-glucan synthase, disrupting fungal cell wall integrity. However, rezafungin’s structural modifications—specifically, adding a choline aminal ether in its cyclic peptide core—provide greater chemical stability and resistance to degradation, with a longer half-life (greater than 130 h) and reduced degradation in serum and buffer solutions [43]. Additionally, it remains active after long periods in solution under light, temperature, and pH stress, making it easier to store and administer than first-generation echinocandins [43]. This stability translates into improved tissue penetration and sustained antifungal activity, making once-weekly dosing feasible [46]. Rezafungin shows broad-spectrum activity against a wide range of fungal pathogens, including species of *Candida* and *Aspergillus*, as well as azole-resistant strains. It is particularly potent against *Candida auris*, *C. albicans*, *C. glabrata*, and *C. tropicalis*, demonstrating similar or superior activity to other echinocandins [47]. This drug is being evaluated in several clinical trials regarding the treatment of candidemia, invasive candidiasis, and aspergillosis [46]. It has been reported that rezafungin shares similar resistance mechanisms with other echinocandins, primarily involving mutations in the *FKS* genes [48]. While these mutations lead to reduced drug binding and decreased efficacy, the high potency of rezafungin helps to mitigate this resistance development [46].

PQA-Az-13: PQA-Az-13 is a newly synthesized hybrid antifungal compound designed to combat *Candida auris*, a multidrug-resistant yeast pathogen that poses a significant public health threat [49]. It has not yet been evaluated on other fungal species and constitutes a unique proposal integrating indazole, pyrrolidine, and arylpiperazine scaffolds with a trifluoromethyl substitution, contributing to its antifungal properties [49]. The compound was evaluated in free and liposomal forms for its efficacy against *C. auris* biofilms, which are critical for the pathogen’s persistence and drug resistance. PQA-Az-13 disrupts biofilm formation and exhibits potent antifungal activity against *C. auris* by affecting amino acid biosynthesis, metabolism, and energy production processes within fungal cells. Proteomic analysis showed that PQA-Az-13 significantly reduces the levels of proteins involved in these pathways, impairing the ability of *C. auris* to maintain biofilms and cellular integrity. Additionally, PQA-Az-13 negatively impacted proteins associated with biofilm regulation, fungal adhesion, and vesicle trafficking—key processes that support the virulence of *C. auris*. It demonstrated strong antifungal activity against 10 *C. auris* isolates from multiple global clades, with minimum inhibitory concentrations (MICs) ranging from 0.67 to 1.25 µg/mL. These values are lower than those observed for fluconazole (2 to >256 µg/mL for all the strains tested) and amphotericin B against certain resistant strains (1.5 to 4 µg/mL for five of the tested strains, particularly pan-drug-resistant isolates), highlighting its potential as an alternative treatment for resistant infections. Due to its hydrophobic nature, PQA-Az-13 was encapsulated in cationic liposomes to improve its solubility, stability, and bioavailability. Liposomes loaded with PQA-Az-13 exhibited high encapsulation efficiency (97.2%) and stability, retaining their size, charge, and antifungal activity over time. The liposomal formulation demonstrated enhanced activity against *C. auris* biofilms in vitro and significantly reduced *C. auris* colonization in an ex vivo pig skin model, achieving an 83% reduction in *C. auris* biofilm growth, outperforming control liposomes. Its efficacy against *C. auris* in biofilm models and its reduced toxicity in dermal applications suggest that it may be useful in systemic and topical treatments for multidrug-resistant fungal infections [49].

AM-2-19 (SF001): A newly developed experimental polyene antifungal optimized for reduced renal toxicity while maintaining strong antifungal potency [50,51]. Unlike traditional polyenes, such as amphotericin B (AmB), which are highly effective but nephrotoxic, AM-2-19 selectively extracts ergosterol from fungal cell membranes without significantly binding cholesterol in mammalian cells, thus reducing its renal toxicity [50]. AM-2-19 operates through a sterol sponge mechanism, forming supramolecular aggregates that selectively extract ergosterol from fungal membranes, disrupting membrane integrity and leading to cell death. This mechanism is similar to AmB but has been modified to increase its specificity for ergosterol over cholesterol, reducing toxicity to human renal cells. The key modification in AM-2-19 is the epimerization of the C2′ stereocenter and modification at C16, which accelerates ergosterol extraction while preventing cholesterol binding [50]. AM-2-19 demonstrates excellent pharmacokinetic properties, allowing for the administration of higher doses without significant nephrotoxicity [50,51]. In mouse models, AM-2-19 showed no significant renal damage, even at doses up to 45 mg/kg, while AmB caused substantial renal injury at much lower doses (2–4 mg/kg). The modifications in AM-2-19 allow for faster ergosterol extraction rates, increasing its antifungal potency while maintaining its renal-sparing properties. AM-2-19 has broad-spectrum antifungal activity, showing potent efficacy against *Candida* and *Aspergillus*, including drug-resistant strains, such as *Candida auris* and *Aspergillus fumigatus* [50]. Its minimal inhibitory concentrations (MICs) are comparable or superior to those of AmB in many fungal species, including resistant strains (e.g., *C. auris* 1 µg/mL AM-2-19, 8–4 µg/mL AmB; *C. glabrata* 0.25–0.5 µg/mL AM-2-19, 1 µg/mL AmB; and *A. terreus* 0.5–2 µg/mL AM-2-19, >16 AmB). In animal models of candidiasis, aspergillosis, and mucormycosis, AM-2-19 demonstrated dose-dependent efficacy, with complete fungal eradication at higher doses. AM-2-19 represents a significant advancement in antifungal therapy due to its renal-sparing profile and potent antifungal activity. It is particularly promising for treating invasive fungal infections in immunocompromised patients, where traditional antifungals, such as AmB, pose substantial risks of nephrotoxicity. Developing a micellar formulation (AM-2-19_DP2K) further enhanced its bioavailability and stability for clinical use [50].

N′-Phenylhydrazides: N′-Phenylhydrazides are a newly synthesized class of antifungal compounds designed to combat fluconazole-resistant strains of *Candida albicans*. A total of 52 different N′-phenylhydrazide derivatives were synthesized and evaluated for their antifungal activity, showing promising results against *C. albicans*, particularly resistant strains [52]. These compounds represent potential lead compounds for the development of new antifungal therapies. The antifungal activity of N′-phenylhydrazides is attributed to the production of reactive oxygen species (ROS) and free radicals within fungal cells. Upon hydrolysis by fungal amidase, these compounds generate phenylhydrazine, which undergoes oxidation and releases ROS, such as superoxide anions and hydroxyl radicals. These ROS disrupt mitochondrial function, impair cellular metabolism, and damage the fungal hyphal morphology, contributing to cell death. Additionally, scanning electron microscopy (SEM) revealed significant structural damage to *C. albicans* hyphae after treatment with the most potent compounds, including folding, wrinkling, and invagination of the cell walls. Among the synthesized N′-phenylhydrazides, several compounds exhibited strong antifungal activity, outperforming fluconazole against fluconazole-resistant *C. albicans* strains. Specifically, compound A11 showed the best inhibitory activity, with MIC80 values of 1.9 µg/mL against *C. albicans* SC5314 and 4.0 µg/mL against strain 4395. Compounds B14 and D5 also demonstrated potent antifungal effects, with total activity index (TAI) values of 2.13 and 2.25, respectively. These compounds were particularly effective in inhibiting biofilm formation, a key factor affecting the pathogenicity and drug resistance of *C. albicans*. The antifungal efficacy of N′-phenylhydrazides was influenced by the substitution patterns on the phenyl rings. Electron-withdrawing groups, such as fluorine, chlorine, and trifluoromethyl groups, enhanced antifungal activity by accelerating the production of free radicals. Conversely, compounds with electron-donating groups, such as methyl and methoxy groups, exhibited lower activity. The introduction of heterocyclic groups in certain compounds, such as C4, with a cyclopropyl substitution, also increased antifungal potency. N′-phenylhydrazides—particularly compounds A11, B14, and D5—hold significant potential as novel antifungal agents. Their ability to inhibit fluconazole-resistant *C. albicans* and target biofilm formation presents a valuable opportunity to address drug-resistant fungal infections. Future research will focus on optimizing these compounds for in vivo applications and evaluating their safety and efficacy in clinical settings [52].

## 3. Drug Repurposing as an Alternative Strategy for the Search for Antifungal Drugs

Drug repurposing involves identifying new uses for existing drugs that have already been approved for other indications. This approach leverages existing safety and pharmacokinetic data, significantly reducing the time required to bring effective treatments to the market. Moreover, repurposed drugs can bypass some stages of clinical trials, making the process more cost-effective and efficient than developing entirely new compounds. A recent and thorough review of the most recent reports on the subject has been published by Gómez-Gaviria et al. [53], in which the authors discussed in detail the repurposing of antibacterials, antivirals, statins, and anti-inflammatory drugs as antifungals. The mechanisms of action of these drugs vary but often involve interfering with fungal cell wall biosynthesis, ergosterol production, or disruption of essential cellular processes, such as mitochondrial function; for example, statins interfere with the biosynthesis of ergosterol in fungal cell membranes, a process analogous to their cholesterol-lowering effects in humans [54,55]. Similarly, some antibiotics exhibit synergistic effects with existing antifungals, enhancing their efficacy against resistant fungal strains. Studies have demonstrated that certain antibiotics, such as colistin, can enhance the efficacy of azole antifungals against *Candida* species, including resistant strains, such as *C. auris* [56,57]. Antivirals, such as lopinavir, which was originally designed for HIV, have also been shown to inhibit biofilm formation in *Candida* species, a critical factor affecting their fungal pathogenicity and drug resistance. Anthelmintics, such as miltefosine, have shown promise against resistant species, like *Sporothrix* and *Cryptococcus*, by disrupting fungal membrane integrity and inducing oxidative stress. Despite the potential benefits, repurposing drugs for antifungal use also poses challenges. The primary issue is that repurposed drugs may not specifically target fungi, which can lead to off-target effects and reduced efficacy. Additionally, continuous use of these drugs may drive the evolution of fungal resistance mechanisms, similar to what has occurred with antibacterial resistance [53]. Furthermore, many repurposed drugs were not originally intended for long-term use, which is often necessary for the treatment of chronic fungal infections. As such, further research into dosing regimens and safety is required.

Niclosamide, a salicylanilide originally developed as an anthelmintic, has emerged as a promising antifungal agent [58]. This drug functions by uncoupling mitochondrial oxidative phosphorylation, leading to a collapse of the mitochondrial membrane potential [59]. This disrupts ATP synthesis and increases ROS, ultimately leading to fungal cell death. It also inhibits critical mitochondrial proteins, such as Mge1, which is involved in protein import, and NDU1, which is essential for biofilm formation in *Candida* species [59]. Additionally, it blocks biofilm formation by *Candida albicans* and *Candida auris* and inhibits the filamentation process in *Candida albicans*—a key virulence factor [59]. It has presented fungicidal activity against *Sporothrix brasiliensis*, *Paracoccidioides brasiliensis*, *Histoplasma capsulatum*, and *Cryptococcus neoformans* [60]. This compound is also active against the dermatophyte *Trichophyton tonsurans*, a causative agent of scalp infections [61]. Its potential application extends to neglected tropical diseases, such as eumycetoma caused by *Madurella mycetomatis*, for which treatment options are limited at present [62].

## 4. Natural Products as a Source of Antifungal Agents

Natural antifungals are compounds derived from plants, bacteria, fungi, and other natural sources [63,64,65,66,67,68,69,70,71,72]. These compounds have evolved to protect their host organisms against pathogenic microbes, including fungi, and have shown potential as therapeutic agents for human fungal infections [64]. Recent advances in genomics, molecular biology, and natural product chemistry have facilitated the discovery of new antifungal compounds from nature. Natural antifungals offer several advantages, including lower toxicity, reduced environmental impact, and the potential to target multiple fungal species simultaneously. Although most research in this line has focused on human fungal pathogens, some compounds have been found to present activities against phytopathogens and pathogens of amphibians [64]. We detail some of the sources of these potential natural antifungals in Table 3.

### 4.1. Marine-Derived Compounds as Potential Antifungal Agents

Marine environments, with their immense biodiversity and extreme conditions, are a key source of bioactive compounds with antifungal properties. Several studies have highlighted the potential of marine-derived secondary metabolites, which have a wide range of structural diversity and unique mechanisms of action that target critical fungal cellular processes [63,64,65]. Some of these marine-derived compounds with antifungal properties are as follows:

- Alkaloids: Marine alkaloids, particularly those isolated from sponges, are of great interest for their antifungal properties. Zamamidine D, isolated from marine sponges, showed potent activity against both *Candida albicans* and *Aspergillus niger*, with a minimum inhibitory concentration (MIC) in the micromolar range [66]. Theonellamide G, derived from the Red Sea Marine Sponge *Theonella swinhoei*, exhibited antifungal activity against *Candida albicans* by targeting ergosterol in fungal membranes, leading to cell lysis [67];
- Polyketides: Polyketides represent a broad class of marine-derived compounds with notable antifungal activity. Dicitrinone E, isolated from marine fungi associated with starfish, has activity against fungal phytopathogens, such as *Colletotrichum gloeosporioides*, significantly inhibiting fungal growth at low concentrations [66,68]. Plakortide F Acid (PFA), isolated from marine sponges, exhibited significant activity against *Candida albicans*, *Cryptococcus neoformans*, and *Aspergillus fumigatus*, disrupting calcium homeostasis and inducing fungal cell death through membrane depolarization [69];
- Peptides: Marine-derived peptides, including cyclic peptides, such as Rhodopeptins isolated from marine bacteria from the genus *Rhodococcus*, have been shown to exhibit strong antifungal activities against resistant strains of *Candida albicans* and *Cryptococcus neoformans* [67,70]. Rhodopeptins, with their unique cyclic lipotetrapeptide structure, have presented activity against fungal pathogens at concentrations as low as 0.00125 mg/mL [70]. The antifungal mechanisms of marine peptides often involve disrupting the fungal membrane or interfering with key cellular processes, such as mitochondrial function, making them highly effective against biofilm-forming fungal species [66];
- Depsispeptides: Marine-derived depsispeptides (alkaloids derived from amino acids and hydroxy acids, which contain both amide and ester bonds) are obtained from algae and exhibit antifungal properties by targeting fungal cell walls. Kahalalides consist of a series of depsispeptides that were first identified from the herbivorous marine mollusks *Elysia* spp. and their algal diet *Bryopsis pennata* and *B. plumosa* (green algae) and have demonstrated significant inhibitory effects against *Candida albicans*, primarily through its interference with fungal cell wall synthesis [64,71]. These compounds provide antifungal activity and offer potential immune-modulating properties, enhancing their therapeutic appeal [65];
- Terpenoids: Marine-derived terpenoids, such as Neothyonidioside isolated from sea cucumbers, have shown antifungal activity by targeting ergosterol in fungal cell membranes, which is critical for maintaining cell integrity [72]. Unlike traditional polyenes, these compounds exhibit unique interactions with ergosterol, offering alternative mechanisms for membrane disruption. Terpenoids are particularly valuable for the development of treatments for azole-resistant fungal species [72].

#### Mechanisms of Action

Marine-derived antifungal compounds exhibit a variety of mechanisms of action that target essential fungal cellular components, including:

- Cell Wall Disruption: Compounds such as Q-Griffithsin, a lectin derived from red algae, disrupt the fungal cell wall by binding to *Candida* cell surface mannan, leading to increased permeability and eventual cell death [73];
- Membrane Disruption: Marine peptides, such as Rhodopeptins, target fungal membranes, causing rapid depolarization and inhibiting ergosterol biosynthesis—a critical component of fungal cell membranes [71];
- Mitochondrial Disruption: Marine-derived peptides, such as Arylamide T-2307, selectively disrupt mitochondrial membrane potential in fungal cells, leading to energy depletion and cell death [26];
- Efflux Pump Inhibition: Some marine compounds, such as unnarmicin A and C, have been shown to inhibit fungal efflux pumps, a key resistance mechanism against azole antifungals. By blocking these pumps, these compounds can be used as adjuvants to restore the effectiveness of traditional antifungals, such as azoles, against resistant fungal strains of *Candida albicans* [74].

### 4.2. Plant-Derived Antifungal Compounds

Due to their broad bioactive properties, plant-derived compounds, particularly essential oils, and secondary metabolites have shown promise as effective antifungal agents [75,76,77,78,79,80,81,82]. They exhibit a variety of mechanisms in their antifungal actions, including disruption of cell wall and membrane integrity, inhibition of biofilm formation, and interference with fungal enzyme activity. These effects might contribute to the broad-spectrum efficacy of these natural products against planktonic and biofilm-associated fungal pathogens. Furthermore, many plant-derived compounds have synergistic effects when combined with conventional antifungals, potentially reducing the dosage required and minimizing adverse effects. Recent examples of these effects are described in the following subsections.

#### 4.2.1. Essential Oils from Cymbopogon Species

Prado et al. [75] tested essential oils from *C. citratus* and *C. nardus* against both planktonic and biofilm forms of *Candida albicans*, with minimum inhibitory concentrations (MICs) as low as 2.44 µg/mL for planktonic cells. These oils exhibited synergistic effects when combined with amphotericin B, reducing the necessary dose of the drug and potentially lowering its associated toxicity [75]. The major bioactive components in *Cymbopogon* essential oils include citronellal, citronellol, and geraniol, which have been shown to inhibit *Candida* biofilm formation by disrupting cell adhesion and aggregation [75].

#### 4.2.2. Essential Oils from Cyperus Species

Another promising source of antifungal agents is essential oils from *Cyperus articulatus* and *Cyperus rotundus* [76]. These oils have been evaluated for their antifungal efficacy against *Aspergillus flavus*, a notorious aflatoxin-producing species that affects crops and food storage. Sabaly et al. [76] reported that essential oils from *C. articulatus*, *C. rotundus*, and *Lippia alba* effectively inhibited the growth of *A. flavus* at concentrations between 100 and 1000 ppm. Among these, the oil from *Lippia alba* was the most effective, completely inhibiting fungal growth at 1000 ppm. The major constituents of these oils included mustakone, caryophyllene oxide, and geranial, which are each known for their antimicrobial properties [76].

#### 4.2.3. Secondary Metabolites from *Arcangelisia flava*

Secondary metabolites derived from *Arcangelisia flava* have also demonstrated potent antifungal activity. Hendra et al. [77] investigated two major compounds—palmatine and fibraurin—extracted from the plant’s root, which exhibited MICs ranging from 15.62 to 62.5 µg/mL against *Candida glabrata* and *Candida krusei*. These metabolites were found to act by inhibiting critical fungal enzymes, such as lanosterol 14-α demethylase, which is essential for ergosterol biosynthesis. Molecular docking studies further supported these findings, showing strong binding affinities of palmatine and fibraurin to fungal enzyme targets [77].

#### 4.2.4. Chromones as Antifungal Agents

Chromones are oxygen-containing benzo-fused γ-pyrone heterocycles that are widely distributed in the plant kingdom [78]. These compounds have garnered considerable interest due to their structural diversity and a broad range of biological activities, including anti-inflammatory, antitumor, and antimicrobial effects. In recent years, chromone derivatives have been extensively studied for their potential as antifungal agents, particularly against *Candida* species, which are responsible for a significant proportion of invasive fungal infections. Lee et al. [78] tested the antifungal properties of 27 commercially available chromone derivatives against nine *Candida* species, including drug-resistant strains, such as *Candida auris* and fluconazole-resistant *Candida albicans*. Among these, four chromone-3-carbonitriles stood out for their high efficacy, with minimum inhibitory concentrations (MICs) ranging from 5 to 50 µg/mL. These compounds were particularly effective against *Candida glabrata*, *Candida parapsilosis*, and *Candida albicans* [78].

The antifungal activity of chromone derivatives is believed to stem from their ability to disrupt critical fungal processes, particularly biofilm formation, including in fluconazole-resistant strains. This is achieved by interfering with the following two major virulence factors:

- Hyphae Formation: Chromones reduced the transition of *C. albicans* from yeast to a hyphal form, a process essential for tissue invasion and biofilm maturation. Hyphal development plays a critical role in biofilm formation and pathogenesis, as the filamentous form of the fungus can penetrate epithelial layers, leading to deeper infections [78];
- Downregulation of Biofilm-Related Genes: Transcriptomic analysis revealed that chromone-3-carbonitriles downregulated key genes involved in biofilm formation, such as TEC1 and UME6 [78]. These genes regulate the morphological transition from yeast to a hyphal form, which is essential for biofilm development. A reduction in the expression of these genes leads to a decrease in hyphal production, limiting the formation of biofilms and thus impairing the fungi’s ability to adhere to surfaces and resist antifungal treatments [78].

Structure–activity relationship (SAR) analysis of the chromones revealed that specific functional groups at the third position of the chromone scaffold significantly enhance their antifungal activity. In particular, the 3-carbonitrile group was critical for potent antifungal activity. Substituents at the sixth position, such as bromine and isopropyl groups, also enhanced the antifungal properties by increasing the hydrophobic interactions between the chromone derivatives and fungal targets, which may contribute to the inhibition of biofilm-related genes and fungal enzymes.

Lee et al. [78] also addressed the safety profile of chromone-3-carbonitriles by conducting toxicity assays using plant and nematode models. Their results demonstrated that these chromone derivatives exhibited mild to no toxicity, making them promising candidates for further drug development. The lack of significant toxicity is crucial for their potential use in clinical settings, particularly for immunocompromised patients at higher risk for severe fungal infections [78].

### 4.3. Bacteria from Amphibian Skin Microbiomes as Sources of Antifungals

Amphibian species have been experiencing significant population declines globally, driven by habitat loss and emerging diseases, such as chytridiomycosis, caused by *Batrachochytrium dendrobatidis* and *B. salamandrivorans*. Interestingly, not all amphibians are equally susceptible to these pathogens. Amphibian skin plays a crucial role in this defense, acting as a barrier and a reservoir of antifungal bacteria. The amphibian skin microbiome is a complex ecosystem where bacteria interact, potentially enhancing antifungal defense against *B. dendrobatidis* and other pathogenic fungi [79,80]. Certain bacteria act as “hub taxa” within these microbial communities, playing key roles in maintaining community stability and functional capacity. For example, *Pseudomonas* and *Acinetobacter* species often form highly interactive networks within amphibian skin microbiomes, facilitating the production of antifungal metabolites. In addition to direct antifungal activity, some bacteria stimulate host immune responses that contribute to fungal defense. Studies using *Arabidopsis thaliana* and *Solanum lycopersicum* infected with *B. cinerea* showed that the application of bacterial isolates from amphibian skin, such as *Acinetobacter* spp. C32I, triggered the plant’s defense mechanisms [81]. These mechanisms involve the activation of salicylic acid (SA) and jasmonic acid (JA) pathways, which are crucial for systemic acquired resistance (SAR) [81]. Studies on salamanders from the Appalachian region have shown that the composition and function of the skin mucosome (a combination of AMPs and skin-associated bacteria) are highly correlated with resistance to chytridiomycosis. In particular, salamander species with higher mucosome diversity and abundance of *B. dendrobatidis* inhibitory bacteria—such as those from the *Pseudomonas* and *Acinetobacter* genera—showed lower Bd infection prevalence [79]. *Janthinobacterium lividum* isolated from salamanders has been shown to produce violacein, a compound that inhibits the growth of *B. dendrobatidis*. Other skin-associated bacteria produce antimicrobial peptides (AMPs) contributing to skin defense [79]. These findings underscore the importance of skin microbiomes in amphibian immunity and their potential role as a source of antifungal compounds.

*Acinetobacter* species isolated from frogs, such as *Agalychnis callidryas* and *Craugastor fitzingeri*, have been identified as potent inhibitors of *B. dendrobatidis* and other fungi, such as *Botrytis cinerea*, a common plant pathogen. Cevallos et al. [80] performed genomic characterization of *Acinetobacter* strains and found that they possess multiple biosynthetic gene clusters (BGCs) responsible for producing antifungal compounds—such as volatile organic compounds (VOCs) and secondary metabolites—that target the cell walls of fungi. These metabolites disrupt membrane integrity, inhibit spore germination, and prevent mycelial growth [80,81].

Further research is needed to explore the full spectrum of antifungal compounds produced by these bacteria and develop effective strategies for their application in diverse environments.

Despite the promising results obtained with natural antifungal agents, several challenges remain. The variability in the composition of natural products, their stability, and the risk of developing resistance are key concerns. However, advances in biotechnology and genomics are providing new tools for optimizing the production and efficacy of natural antifungals. Using genomics and bioinformatics to explore the genomes of antifungal-producing organisms has accelerated the discovery of new bioactive compounds; for example, the genome of *Bacillus velezensis* has revealed multiple biosynthetic gene clusters responsible for the production of antifungal compounds [82]. Such genomic insights can guide the development of more targeted and effective antifungal therapies. Bioengineering approaches, such as modifying microbial strains to enhance the production of antifungal compounds, hold great promise for the future. Synthetic biology techniques can engineer bacteria and fungi to produce higher yields of antifungal compounds, thereby increasing their efficacy and scalability.

## 5. Nanoparticles and Green Synthesized Materials as Antifungal Compounds

Nanotechnology offers a promising platform, particularly through the development of nanoparticles (NPs) with antimicrobial properties [83,84,85,86,87]. The green synthesis of nanoparticles using plant extracts and biocompatible materials represents an eco-friendly and sustainable approach to the production of these agents.

### 5.1. Magnetic Nanoparticles (MNPs)

Magnetic nanoparticles (MNPs), specifically Fe_3_O_4_-based nanostructures, have demonstrated significant antifungal activity, particularly when functionalized with other bioactive compounds. Azadi et al. [83] explored the antifungal activity of Fe_3_O_4_@SiO_2_/Schiff-base/Cu(II) magnetic nanoparticles against *Candida* species. The Schiff-base complexes, in combination with copper ions, disrupted fungal cell membranes, leading to increased reactive oxygen species (ROS) generation, which ultimately caused fungal cell death. The Fe_3_O_4_@SiO_2_ core–shell structure enhanced stability and provided a high surface area for better interaction with fungal cells [83]. The minimum inhibitory concentration (MIC) values due to these nanoparticles ranged from 8 to 64 µg/mL, with *C. parapsilosis* being the most susceptible. This highlights the potential of MNPs as broad-spectrum antifungal agents, especially in combating drug-resistant fungal strains.

### 5.2. Silver Nanoparticles (AgNPs)

Silver nanoparticles (AgNPs) have been studied extensively for their antifungal properties, and the green synthesis of AgNPs using plant extracts provides an environmentally friendly alternative to traditional chemical methods. Di Muzio et al. [84] utilized a green synthesis approach to produce AgNPs embedded in gellan gum-based nanocomposite films using kiwifruit peel extract as a reducing agent [84]. The size of AgNPs produced through this method ranged from 10 to 20 nm, and the particles were very stable and presented strong antifungal properties against multiple *Candida* species, including *C. albicans*, *C. glabrata*, and *C. krusei*.

A primary mechanism by which AgNPs exert their antifungal effects is through the disruption of fungal cell membranes. The nanoparticles interact with the lipid bilayer of the fungal membrane, leading to increased membrane permeability and, ultimately, cell death. Ahamad et al. [85] reported that AgNPs synthesized from *Anabaena variabilis* (a filamentous cyanobacterium) caused significant membrane permeabilization in *C. albicans* at concentrations as low as 25 µg/mL, with 79% cell permeability and 62.5% biofilm inhibition [85]. The small size of nanoparticles allows them to penetrate cell walls, disrupting the membrane’s integrity and leading to cellular collapse. They also reported that AgNPs effectively inhibited *C. albicans* biofilms, with a minimum inhibitory concentration (MIC) of 12.5 µg/mL [85]. Disruption of the yeast-to-hypha transition was also noted in this study, further highlighting the potential of AgNPs in treating candidiasis.

Silver nanoparticles also induce oxidative stress in fungal cells by generating reactive oxygen species (ROS). These ROS can damage cellular components such as proteins, lipids, and DNA, leading to cell apoptosis. AlJindan and AlEraky [86] demonstrated that AgNPs caused significant ROS generation in *Candida auris*, contributing to the strong antifungal activity of the nanoparticles. The level of ROS generation appears to be dose-dependent, with higher concentrations of AgNPs resulting in greater oxidative damage to fungal cells. *C. auris* is known for its resistance to multiple antifungal drugs, and the authors also evaluated the antifungal and antibiofilm activity of the AgNPs against clinical isolates of *C. auris*, reporting MIC values of <6.25 µg/mL and Minimal Fungicidal Concentration (MFC) values ranging from 6.25 to 12.5 µg/mL. These results suggest that AgNPs could be an alternative treatment option for infections caused by multidrug-resistant *C. auris*, particularly in hospital settings, where outbreaks are common.

Biofilm formation significantly contributes to the pathogenicity and drug resistance of fungal species such as *C. albicans* and *C. auris*. Biofilms protect fungal cells from antifungal agents and the host immune system. AgNPs have been shown to inhibit both biofilm formation and disrupt pre-formed biofilms. Vazquez-Munoz et al. [87] reported that silver nanoparticles were highly effective against biofilms of *Candida auris*. The Medium Inhibitory Concentration (IC50) values for biofilm inhibition ranged from 1.2 to 6.2 µg/mL, demonstrating potent antibiofilm activity [87]. AgNPs interfere with the adhesion and maturation stages of biofilm formation, reducing the pathogen’s ability to form protective biofilms on medical devices and other surfaces.

## 6. Emerging Antifungal Targets and Innovative Therapeutic Strategies

Antifungal therapies target ergosterol biosynthesis, cell wall integrity, and nucleic acid synthesis. However, the emergence of resistant fungal strains demands the exploration of novel targets and approaches. Emerging antifungal targets include fungal biofilms, cell wall enzymes, virulence factors, and novel small molecules, complemented by innovative therapeutic strategies to combat fungal infections.

### 6.1. Cell Wall as a Target for Antifungal Development

#### 6.1.1. Cell Wall Integrity and Mannosidases

The fungal cell wall is a key structure for maintaining fungal integrity and virulence, making it an attractive target for antifungal drugs. The *C. albicans* mannosidases Dfg5 and Dcw1 both encode cell wall GPI-anchored mannanase/mannosyltransferase enzymes (gh-76 family), playing critical roles in cell wall protein integration and are required for the maintenance of cell wall integrity and hyphal morphogenesis. These enzymes are involved in the covalent cross-linking of cell wall glycoproteins and impact virulence by contributing to hyphal formation, a key pathogenic feature of *C. albicans* [88]. Recent studies have shown that mutations in *dfg5* and *dcw1* lead to defective cell wall assembly, rendering the fungal cells susceptible to antifungal drugs. Furthermore, these enzymes regulate chitin synthesis, with their disruption resulting in defects in septum formation and cell division [88].

#### 6.1.2. Chitin Synthases

Chitin, an essential component of the fungal cell wall, is synthesized by chitin synthases (Chs). *C. albicans* has multiple chitin synthases (Chs1, Chs2, Chs3, and Chs8), and these enzymes work in coordination to maintain cell wall integrity. Chitin synthases represent a promising target for antifungal development due to their essential role in fungal viability. Disrupting chitin biosynthesis through inhibitors of chitin synthases impairs fungal growth, providing a potential avenue for novel antifungal agents [89]. Polyoxins and nikkomycins (peptide nucleosides isolated from *Streptomyces* spp.) are the two antifungals developed to date that interfere with chitin synthesis by inhibiting the chitin synthases in vitro [89]. Nikkomycin exhibited some in vitro efficacy against *Coccidioides immitis* and *Blastomyces dermatitidis* [90]; however, its effectiveness largely depends on its synergistic effects with echinocandins, particularly for *C. albicans* and *A. fumigatus* [91,92]. Meanwhile, polyoxins are mostly ineffective or fungistatic for *C. albicans*, *C. immitis*, and *C. neoformans* [93].

### 6.2. Biofilm Inhibition

Biofilm formation is a major virulence factor for fungal pathogens, particularly in *C. albicans*. Biofilms confer resistance to antifungal treatments by forming protective extracellular matrices and reducing the efficacy of antifungal drugs. Thus, inhibiting biofilm formation has emerged as a novel antifungal strategy. Pierce et al. [94] identified a novel series of diazaspiro–decane structural analogs capable of inhibiting *C. albicans* biofilm formation without affecting fungal growth [94]. These compounds represent antivirulence agents that target biofilm structure and prevent fungal colonization of surfaces such as medical devices.

Biofilm inhibitors can be combined with traditional antifungals to enhance their efficacy. For example, disrupting biofilm formation through the use of small molecules can improve drug penetration, allowing conventional antifungals to reach fungal cells embedded within biofilms [94].

### 6.3. Inhibition of Hyphal Morphogenesis

Hyphal morphogenesis is a critical virulence factor for *C. albicans*, facilitating tissue invasion and biofilm formation. Romo et al. [95] developed a series of small molecules that specifically inhibit the yeast-to-hypha transition in *C. albicans*, effectively reducing its pathogenicity. These molecules target the signaling pathways that control hyphal development, including the cAMP and MAPK pathways, preventing filamentation and reducing biofilm formation. The lead compound, N-[3-(allyloxy)-phenyl]-4-methoxybenzamide, demonstrated in vivo efficacy in murine models of invasive candidiasis [95]. By targeting virulence factors such as hyphal morphogenesis, these molecules represent an innovative antifungal approach that reduces fungal pathogenicity without promoting drug resistance.

### 6.4. Targeting Fungal Mitochondrial Function

Mitochondria play key roles in energy production and metabolic regulation in fungal cells, making them a viable target for antifungal therapy. Targeting mitochondrial function can disrupt fungal bioenergetics and induce cell death. In addition to T-2307 (see Section 2.2), isothiocyanates—particularly benzyl isothiocyanate (BITC) isolated from *Carica papaya* L. seeds and cruciferous vegetable species—have been shown to inhibit mitochondrial oxidative phosphorylation or disrupt the fungal mitochondrial membrane potential, presenting promising antifungal action [96,97,98]. Targeting fungi-specific mitochondrial processes may reduce the risk of toxicity in humans while effectively combating fungal infections [96].

### 6.5. Inhibition of Heat Shock Protein (Hsp90)

Heat shock protein 90 (Hsp90) is a molecular chaperone that regulates fungal stress responses and certain antifungal resistance mechanisms. Inhibiting the function of Hsp90 sensitizes fungal cells to antifungal drugs, making it a promising target for overcoming resistance. Hsp90 inhibitors have been shown to enhance the efficacy of azoles and echinocandins, particularly against drug-resistant strains of *C. albicans* and *Aspergillus fumigatus* [99,100]. By disrupting fungal stress tolerance, Hsp90 inhibitors reduce the survival of fungal cells under drug-induced stress [99,100].

## 7. Artificial Intelligence in the Study of Antifungal Treatments, Diagnostics of Mycoses, and Prediction of Drug Resistance

In recent years, artificial intelligence (AI) has revolutionized biomedical research, providing unprecedented tools and techniques to address complex challenges in the healthcare domain [101,102,103]. Effective managing fungal infections is complicated by diagnostic difficulties, limited treatment options, and rising antifungal resistance [101]. AI offers transformative potential for discovering antifungal agents, improving diagnostics, and predicting drug resistance, potentially reducing the morbidity and mortality associated with mycoses [102].

### 7.1. AI in the Discovery of Antifungal Agents

The discovery of antifungal agents has traditionally relied on labor-intensive and time-consuming laboratory work. AI techniques, particularly machine learning (ML), offer an alternative approach by efficiently analyzing large datasets, predicting active compounds, and optimizing drug candidates [101]. In such an approach, algorithms are trained on known antimicrobial compounds, learning the structural and physicochemical properties associated with their activities. Once trained, these models can predict the efficacy of untested compounds, significantly accelerating the drug discovery process.

Additionally, generative models—including deep generative adversarial networks (GANs)—have shown promise in de novo drug design by creating novel molecular structures with desired properties [102]. These methods can generate objects with specific characteristics, such as efficacy against a particular target, making them ideal for discovering potential drug candidates. If trained on antifungal pharmacophores, such models could potentially generate candidate molecules with increased specificity and reduced toxicity. Stokes et al. [103] reported the discovery of a novel antibacterial compound using a deep learning model, a methodology that can be similarly applied to antifungals. These AI-driven methods have yielded several promising candidates for further validation and could be used to expand the antifungal drug repertoire significantly. Resources such as the Protein Data Bank (PDB) and advanced predictive tools such as Al-phaFold 3 (AF3) are expected to enrich the structural biology of antifungal targets [104]. These technologies will facilitate the stepwise modeling of protein–ligand interactions, which is important for drug discovery [105,106]. The power of AF3’s diffusion-based architecture lies in its ability to accurately predict complex biomolecular interactions, surpassing that of traditional docking methods for the prediction of binding poses and affinities, including for difficult targets [104,106].

### 7.2. AI in Diagnostics of Mycoses

Diagnosing mycoses remains challenging due to the often non-specific symptoms of fungal infections and the limitations of traditional diagnostic methods. While informative, microscopy, culture techniques, mass spectrometry, and serological tests are labor-intensive and may lack sensitivity or specificity, particularly for rare or emerging fungal pathogens. AI-based diagnostics, particularly those leveraging image analysis and deep learning, provide a valuable alternative by enabling the rapid, accurate, and automated identification of fungal pathogens [107,108].

AI-driven image recognition systems have been developed for diagnostic microscopy, allowing for the automated detection of fungi in clinical samples with high accuracy and high training speed (2.4 h in contrast to months or even years for a human technician to be properly trained) [107]. Convolutional neural networks (CNNs), a deep learning model, have shown promise in analyzing complex image data from histopathological slides, distinguishing fungal cells from host cells with minimal human intervention. By training on large datasets of microscopic images, CNNs can learn to identify specific morphologies and biomarkers associated with different fungal species, offering a valuable diagnostic tool for clinical mycology laboratories. Bermejo-Peláez et al. [108] recently used AI in the form of a mobile app that was able to read cryptococcal antigen (CrAg) and lateral flow assay (LFA) for the diagnosis of cryptococcosis.

### 7.3. AI in Predicting Fungal Drug Resistance

The rapid evolution of antifungal resistance presents a severe challenge regarding effective treatment, particularly for pathogenic fungi such as *Candida auris*, *Aspergillus fumigatus*, and *Cryptococcus neoformans*. Predicting resistance patterns and understanding the underlying mechanisms are essential for guiding treatment and limiting the spread of resistance. AI-based predictive modeling has emerged as a critical tool in this domain, utilizing genetic, phenotypic, and environmental data to forecast drug resistance.

AI algorithms have been developed to analyze genomic data, identifying mutations and other genetic markers associated with antifungal resistance. Machine learning models can be trained on datasets containing resistant and susceptible fungal strains, allowing them to detect resistance-associated features. In their study, Li et al. [109] applied ML to genomic data from *Candida auris* strains, identifying resistance markers to fluconazole, itraconazole, voriconazole, and micafungin. This approach facilitates the development of rapid, personalized resistance diagnostics that can predict a pathogen’s likelihood of resistance before treatment is administered. These predictive models are invaluable for the development of surveillance strategies, as they allow researchers and healthcare professionals to anticipate outbreaks of resistant strains and implement pre-emptive measures.

## 8. Conclusions

This review highlights both the significant advancements and persistent challenges in the field of antifungal therapy. Despite the increase in antifungal research, recent efforts have predominantly targeted a limited range of fungal pathogens, focusing on prominent pathogens such as *Candida*, *Aspergillus*, and *Cryptococcus*. While critical for addressing the widespread and resistant infections caused by these organisms, this emphasis leaves other medically important pathogens underexplored. Emerging fungal pathogens, including *Paracoccidioides*, *Histoplasma*, *Coccidioides*, and *Pneumocystis*, among others, continue to pose serious health risks in endemic regions yet are underserved by current antifungal development pipelines.

The increasing incidence and resistant fungal infections, notably with respect to *Candida auris* and *Aspergillus fumigatus*, underscores the urgent need for innovative therapeutic strategies. The exploration of new antifungal agents, including next-generation compounds, such as K21, olorofim, VT-1161, T-2307, and ibrexafungerp, demonstrates the field’s response to these challenges. These agents, with diverse mechanisms targeting fungal cell walls, ergosterol biosynthesis, and mitochondrial function, provide promising alternatives to current therapies limited by resistance, toxicity, and efficacy (Figure 1).

In addition to synthetic compounds, this review emphasizes the potential of natural antifungal products and drug repurposing as viable alternatives. Natural sources, including marine- and plant-derived compounds, offer unique mechanisms of action, such as cell wall and membrane disruption, which could mitigate resistance risks. Additionally, the repurposing of existing drugs—particularly those with established safety profiles—represents an efficient pathway to expand the therapeutic options for resistant infections.

Furthermore, nanotechnology and bioengineering are poised to augment the efficacy of antifungal treatments by enabling targeted drug delivery and enhanced bioavailability. These approaches may significantly improve treatment outcomes, especially in those immunocompromised patients who are most susceptible to invasive mycoses.

Despite its promising applications, the integration of AI into antifungal research faces several challenges. Data quality and availability are critical constraints, as ML models require extensive, high-quality training datasets to function accurately. In many regions, mycoses are under-reported, and the availability of comprehensive fungal genomic and phenotypic datasets is limited. Moreover, while AI models can suggest antifungal candidates or predict resistance, experimental validation remains essential, emphasizing the need for close collaboration between computational and laboratory research.

Future research should prioritize the optimization of antifungal combinations, antivirulence strategies, and clinical evaluation of emerging therapies. The integration of these approaches, together with robust diagnostic methods for pathogen-specific treatments, holds the potential to transform antifungal therapy. The spectrum of antifungal development should also be expanded beyond the currently targeted fungi, encompassing pathogens such as *Paracoccidioides*, *Histoplasma*, *Coccidioides*, and *Pneumocystis*. These advances are essential to address the dynamic landscape of fungal infections, improve patient outcomes, and reduce the global burden of fungal diseases.

## Figures and Tables

**Table 1:** Major classes of antifungal agents and their characteristics.

Class	Mechanism of Action	Use	Limitations
Polyenes	Binds ergosterol, creates membrane pores	Broad-spectrum, *Candida*, *Aspergillus*	High toxicity (nephrotoxicity)
Azoles	Inhibits ergosterol synthesis	Broad-spectrum, *Candida*, *Aspergillus*	Resistance, drug interactions
Echinocandins	Inhibits β-(1,3)-glucan synthase	*Candida*, *Aspergillus*	Ineffective against some species, emerging resistance
Flucytosine	Interferes with RNA and DNA synthesis	*Cryptococcus* in combination therapy	Rapid resistance, bone marrow toxicity

**Table 2:** Antifungals (both in use and candidates) and their known and proposed mechanisms of action.

Compound	Class	Mechanism of Action	Key Antifungal Activity	Clinical Relevance
K21	Silica Quaternary Ammonium Compound	Membrane disruption, leading to fungal cell lysis	Effective against fluconazole-resistant *Candida* species, including *C. albicans*, *C. glabrata*, and *C. dubliniensis*	Synergistic with fluconazole; potential for HIV-associated candidiasis
Olorofim (F901318)	Orotonomide	Inhibits DHODH, blocking pyrimidine biosynthesis essential for nucleic acids and cell wall synthesis	Active against *Aspergillus fumigatus* and other filamentous fungi, causing changes in chitin content, vacuolar swelling, and inhibition of mitosis	Promising for treatment of invasive fungal infections, particularly resistant aspergillosis
VT-1161	Tetrazole	Inhibits lanosterol 14α-demethylase, disrupting ergosterol synthesis	Potent against *Candida glabrata* and *Candida krusei*, including strains resistant to azoles	Targets resistant *Candida* strains and may require combination therapies
T-2307	Arylamidine	Disrupts mitochondrial membrane potential, leading to fungal cell death	Highly effective against *Candida tropicalis*, particularly in biofilm inhibition and eradication	Novel mitochondrial-targeting mechanism; low cytotoxicity, potential clinical use
Ibrexafungerp	Triterpenoid	Inhibits β-(1,3)-D-glucan synthase, disrupting fungal cell wall synthesis	Broad-spectrum activity against *Candida* species, including echinocandin-resistant strains; high bioavailability in vaginal tissues	FDA-approved for vulvovaginal candidiasis (VVC) and recurrent VVC
Fosmanogepix	First-in-Class GPI Anchor Inhibitor	Inhibits Gwt1, affecting GPI-anchored mannoproteins in fungal cell walls	Broad-spectrum activity, including against *Candida auris*, *Aspergillus fumigatus*, and rare molds, such as *Scedosporium*	Effective in resistant fungal infections, including rare and difficult-to-treat molds
Rezafungin	Echinocandin	Inhibits β-(1,3)-D-glucan synthase, disrupting cell wall integrity	Long half-life; broad-spectrum activity against *Candida* and *Aspergillus*, including biofilm-associated infections and resistant strains	Once-weekly dosing; potential for invasive candidiasis and aspergillosis
PQA-Az-13	Hybrid Antifungal	Disrupts biofilm formation by affecting amino acid biosynthesis and metabolism	Strong activity against *Candida auris* biofilms; enhanced in liposomal formulations	Suitable for resistant biofilm infections; potential for topical and systemic use
AM-2-19	Polyene	Selectively extracts ergosterol from fungal membranes, reducing renal toxicity	Broad-spectrum activity against *Candida*, *Aspergillus*, and drug-resistant strains, such as *C. auris*; renal-sparing properties	Reduced nephrotoxicity compared to amphotericin B; potential for invasive fungal infections
N′-Phenylhydrazides	Phenylhydrazide	Induces ROS production and mitochondrial disruption, leading to fungal cell death	Potent against fluconazole-resistant *Candida albicans* and biofilm formation; enhanced activity with electron	Potential for treating drug-resistant *Candida* infections, especially those with biofilms
Niclosamide	Repurposed Anthelmintic	Disrupts mitochondrial oxidative phosphorylation, leading to fungal cell death	Broad-spectrum activity, including against *C. auris* and filamentation in *Candida albicans*; effective against *Sporothrix*, *Histoplasma*, *Cryptococcus*, and *Trichophyton*	Potential for use in both superficial and systemic fungal infections

**Table 3:** Potential use of natural products for antifungal compound development with different antifungal mechanisms.

Source	Compound Types	Examples	Antifungal Mechanism	Target Fungi
Marine Environments	Alkaloids	Zamamidine D, Theonellamide G	Disrupts fungal membrane via ergosterol binding	*Candida albicans*, *Aspergillus niger*
	Polyketides	Dicitrinone E, Plakortide F Acid	Interferes with calcium homeostasis	*Candida albicans*, *Cryptococcus neoformans*
	Peptides	Rhodopeptins	Membrane disruption and mitochondrial inhibition	*Candida albicans*, *Cryptococcus neoformans*
Terrestrial Plants	Essential Oils	Cymbopogon (Lemongrass), Cyperus species	Inhibits biofilm formation, membrane disruption	*Candida albicans*, *Aspergillus flavus*
	Secondary Metabolites	Arcangelisia flava	Inhibits fungal enzymes	*Candida* spp.
Amphibian Skin Microbiome	Bacterial-derived Compounds	Violacein from *Janthinobacterium lividum*	Inhibits fungal cell wall synthesis	*Batrachochytrium dendrobatidis*

## Data Availability

No data generated.
